# Nuclear and cytoplasmic poly(A) binding proteins (PABPs) favor distinct transcripts and isoforms

**DOI:** 10.1093/nar/gkac263

**Published:** 2022-04-19

**Authors:** Angela L Nicholson-Shaw, Eric R Kofman, Gene W Yeo, Amy E Pasquinelli

**Affiliations:** Division of Biology, University of California, San Diego, La Jolla, CA 92093, USA; Department of Cellular and Molecular Medicine, University of California San Diego, La Jolla, CA 92093, USA; UCSD Stem Cell Program, Sanford Consortium for Regenerative Medicine, La Jolla, CA 92037, USA; Institute for Genomic Medicine, University of California San Diego, La Jolla, CA 92093, USA; Department of Cellular and Molecular Medicine, University of California San Diego, La Jolla, CA 92093, USA; UCSD Stem Cell Program, Sanford Consortium for Regenerative Medicine, La Jolla, CA 92037, USA; Institute for Genomic Medicine, University of California San Diego, La Jolla, CA 92093, USA; Division of Biology, University of California, San Diego, La Jolla, CA 92093, USA

## Abstract

The poly(A)-tail appended to the 3′-end of most eukaryotic transcripts plays a key role in their stability, nuclear transport, and translation. These roles are largely mediated by Poly(A) Binding Proteins (PABPs) that coat poly(A)-tails and interact with various proteins involved in the biogenesis and function of RNA. While it is well-established that the nuclear PABP (PABPN) binds newly synthesized poly(A)-tails and is replaced by the cytoplasmic PABP (PABPC) on transcripts exported to the cytoplasm, the distribution of transcripts for different genes or isoforms of the same gene on these PABPs has not been investigated on a genome-wide scale. Here, we analyzed the identity, splicing status, poly(A)-tail size, and translation status of RNAs co-immunoprecipitated with endogenous PABPN or PABPC in human cells. At steady state, many protein-coding and non-coding RNAs exhibit strong bias for association with PABPN or PABPC. While PABPN-enriched transcripts more often were incompletely spliced and harbored longer poly(A)-tails and PABPC-enriched RNAs had longer half-lives and higher translation efficiency, there are curious outliers. Overall, our study reveals the landscape of RNAs bound by PABPN and PABPC, providing new details that support and advance the current understanding of the roles these proteins play in poly(A)-tail synthesis, maintenance, and function.

## INTRODUCTION

The vast majority of eukaryotic RNAs transcribed by RNA Polymerase II have a 3′ poly(A) tail added to them co-transcriptionally. The poly(A) tail serves a wide range of functions - promoting nuclear export (1), protecting the RNA from exonucleases and degradation (2,3), and enhancing translation (2,4,5). These roles are largely mediated through RNA-binding proteins. The nuclear and cytoplasmic poly(A) binding proteins (PABPs), PABPN and PABPC, are the main poly(A) binding proteins and have distinct roles when they are bound to the tail (6).

PABPN1 (hereafter referred to as PABPN) is involved in the initial creation of the poly(A) tail in the nucleus. Once cleavage occurs near the poly(A) site (PAS) of a nascent RNA, poly(A) polymerase (PAP) begins synthesizing the tail (7,8). After PAP has added 11–14 adenosines, PABPN is then able to bind to the growing tail, which causes PAP to switch to processive synthesis, rapidly completing creation of the tail (9,10). Cleavage and polyadenylation can occur either before or after splicing is fully complete (11–13). PABPN-oligo(A) binding equilibrium measurements determined that the minimum tail length that PABPN can bind is 11 adenosines and the site size covered by PABPN is 11–15 adenosines (14,15). Polyadenylation proceeds until the tail has ∼200–250 adenosines (16–18). The exact mechanism of tail length control is not clear, but PABPN forms a 21 nm spherical particle with poly(A) that is thought to serve as a molecular ruler (19). Although PABPN can contiguously bind along the poly(A) tail, PABPN–PABPN interactions show weak cooperativity (14,19).

When an RNA is fully mature, it can be exported out to the cytoplasm, where PABPC is the predominant protein coating poly(A) tails. The transition from PABPN to PABPC on the tail is not well understood. Reporter studies have suggested that translation may facilitate this transition (20). PABPC supports numerous protein interactions that promote translation and stability such as binding to the translation initiation factor eIF4G and the translation termination factor eRF3 (21–28). PABPC can sequentially bind the poly(A) tail and has a footprint of ∼20–30 adenosine nucleotides (29,30), requiring a minimum of 11 or 12 adenosines in order to bind (31).

Humans have one nuclear PABP, PABPN1, and five cytoplasmic PABP proteins (6,32,33). The major PABPC present in somatic tissues is PABPC1. The four remaining proteins are testis-specific PABP (tPABP, also termed PABPC3), embryonic PABP (ePAB), X-linked PABP (PABPC5), and a PABP discovered as an inducible protein in stimulated T-cells (PABPC4, also known as iPABP) (32,34–38). As PABPC1 is the most abundant cytoplasmic PABP, we focused our studies on PABPC1 and it is referred to throughout the rest of the text as PABPC. Estimates of cellular abundance of PABPN are 2–4 × 10^6^ molecules per cell and for PABPC, 8 × 10^6^ molecules (39,40). Affinities for the poly(A) tail as measured through dissociation constants are similar: 2 nM for PABPN (41) and 0.69 nM-7 nM range for PABPC (40,42,43). Despite sharing a binding substrate, PABPN and PABPC are structurally and functionally distinct from one other. In addition to their predominant localization in the nucleus or cytoplasm respectively, PABPN and PABPC are shuttling proteins, existing in lower abundance in the opposite compartment as well (44–47). The presence of one PABP on the poly(A) tail is not known to exclude the other and therefore PABPN and PABPC may coexist on a single RNA (47).

Although poly(A) tails can reach a full-length size of over 200 adenosines, their length at steady state is much shorter. Early bulk poly(A) studies revealed shorter cytoplasmic lengths (16,48). With the advent of high-throughput sequencing, a few genome-wide studies have confirmed this in human cells (49–52). However, sequencing of poly(A) tails when they are first made has been limited, leaving the question of whether all tails on all transcripts follow these model patterns (53).

In the present study, we provide in depth analysis of the transcripts associated with PABPN and PABPC at steady state in human cells. We find that distinct sets of messenger RNA (mRNA) and non-coding RNA (ncRNA) predominantly bind PABPN or PABPC. Using Ribo-STAMP (54), we present evidence that when transcripts are still associated with PABPN they make contact with the ribosome but less frequently than when they are bound by PABPC. Through Nanopore direct RNA sequencing, we show that distinct isoforms of RNA, differentiated by poly(A) tail length and intron presence, are bound to PABPN or PABPC. Overall, our results capture the broad landscape of RNAs that associate with PABPN and PABPC, providing new insights and confirming some aspects of the current model for PABPN binding nascent polyadenylated transcripts and the accumulation of well-translated mRNAs containing pruned poly(A) tails with PABPC in the cytoplasm.

## MATERIALS AND METHODS

### Cell culture

HEK293T cells were cultured as recommended by the manufacturer, in Dulbecco's modified Eagle's medium (DMEM, Gibco #11965092) supplemented with 10% fetal bovine serum (Gibco #10437-028) and 1% penicillin/streptomycin (Gibco). Cells were grown to 80–85% confluency before collecting for experimental analysis.

### Formaldehyde crosslinking

Ten percent formaldehyde stock solution was made by heating paraformaldehyde to crack it and storing single-use aliquots at –20°C until ready to use. Cells were washed twice on the plate with phosphate-buffered saline (PBS) and then collected into conical tubes with PBS and spun down. Cell pellets were resuspended in 0.1% formaldehyde in PBS and incubated at room temperature for 10 min. Glycine was added to a final concentration of 0.17 mM to quench the formaldehyde and incubated for 5 min. Cells were then spun down and washed with PBS two times before pellets were snap frozen on liquid nitrogen for storage at –80°C.

### siRNA transfection

Knockdowns to validate antibodies used in immunoprecipitations were performed with 200 μM small interfering (si)RNAs targeting either luciferase (control), PABPN or PABPC, using siLentFect (Bio-Rad) transfection reagent according to manufacturer's recommendations at 72 and 24 h before harvest. siRNA duplex oligos ordered from Dharmacon were as follows: siLuciferase: CGUACGCGGAAUACUUCGAUU; siPABPN: GUAGAGAAGCAGAUGAAUA; and siPABPC: GAAAGGAGCUCAAUGGAAA. siPABPC and siPABPN were previously validated and published (55).

### RNA immunoprecipitation

Cell pellets were resuspended in ice-cold RIPA buffer (Thermo Fisher) supplemented with 40 U/ml rRNAseIn Plus (Promega), 0.5 mM DTT, 5 mM EDTA and 1 tablet/25 ml mini cOmplete protease inhibitor cocktail (Roche). Cells were sonicated three times at 8 W: 20 s on, 2 min off, in an ice bath. Lysates were spun down and Protein G Dynabeads (Invitrogen) were prepared to pre-clear the lysates by washing with RIPA buffer twice. Pre-clearing was performed on the nutator for 30 min at 4°C. After this, pre-cleared lysates were incubated with 5 μg antibody for 2 h at 4°C on nutator (anti-PABPC, ab21060 Abcam; anti-PABPN, [EP3000Y] ab75855 Abcam; isotype control anti-rabbit IgG monoclonal ab172730 Abcam; isotype control anti-rabbit IgG polyclonal ab171870 Abcam). Protein G Dynabeads were again prepared by washing with RIPA buffer and 100 μl slurry was added per IP, rotating on nutator for 1 h at 4°C. IPs were washed four times with supplemented RIPA buffer. Final beads were eluted in elution buffer (50 mM Tris–HCl pH 7.2, 5 mM EDTA, 1% SDS, 10 mM DTT) and proteinase K (NEB). Reverse crosslinking was performed on the thermomixer shaking at 1200 rpm, first at 60°C for 20 min to allow proteinase K to work, and then at 70°C for an additional 25 min. RNA was isolated using a standard Trizol (Life Technologies) RNA extraction.

### Illumina library prep

For total cell lysate IPs, cDNA libraries from three independent biological replicates were prepared from 1ug RNA using Illumina ribodepleted RNA stranded kit. Libraries were sequenced on the Illumina HiSeq 4000, single-end reads (75 nucleotides).

For nuclear and cytoplasmic fractionation, cDNA libraries from two independent biological replicates were prepared from 400ng RNA using Illumina ribodepleted RNA stranded kit. Libraries were sequenced on the Illumina NovaSeq 6000, paired-end reads (100 nucleotides).

For RPS2-STAMP IPs, cDNA libraries from three independent biological replicates were prepared from 200 to 300 ng RNA using Illumina ribodepleted RNA stranded kit. Libraries were sequenced on the Illumina NovaSeq 6000, paired-end reads (100 nucleotides).

### Illumina RNA-seq analysis

For total lysate IPs, libraries were at least 44 million reads per sample, with an average of 55 million reads. Average percent of uniquely mapped reads was 88%. For fractionations, libraries were at least 28 million reads per sample, with an average of 29 million reads. Average percent of uniquely mapped reads was 82%. For STAMP experiments, libraries were at least 26 million reads per sample, with an average of 32 million reads. Average percent of uniquely mapped reads was 77%. All reads were aligned to the human genome hg38 primary assembly using STAR. Bam files were sorted and indexed using samtools. featureCounts version 2.0.2 was used to annotate reads, using the flag - -minOverlap 20 and a custom GTF file derived from gencode v34 as described in results (56). Differential expression was calculated using DESeq2 (57).

Due to the contribution of intronic reads to our datasets, calculation of TPM (Transcript per kilobase million) values was performed by separately determining TPMs for exonic regions and intronic regions and then summing together. This was important in order to not skew values by using the full length of the unspliced gene, as introns are very long in comparison to exons. Additionally, for genes that had a large number of intronic reads, using the spliced exonic length would also not be appropriate, as that gene would appear to be more highly expressed than it was.

### Upstream transcription problem genes

Upstream intergenic regions were extracted using bedtools flank -l 2000 -r 0 -s. Intervening upstream genes that had any overlap with this region were removed with bedtools subtract. Reads that did not align to intronic or exonic regions were extracted from bam files with fgrep. Coverage across the 2000 bp upstream region was determined with bedtools coverage -S -split. A TPM value was determined for this upstream region and compared to the TPM of the adjacent downstream gene. If a ratio of 20% of the signal (determined by TPM) was present in the upstream region, and a breadth of 70% of that region was covered, that gene was determined to have significant enough upstream transcription so as to not be a reliable functional coding product, and was removed from subsequent analysis. Genes that showed this pattern in either the input or IP condition were both removed from that IP analysis.

### Western blotting

Western blotting was performed as previously described (58,59) using the following antibodies: Calreticulin, Cell Signaling 2891; Histone H3, abcam ab1791; U1 snRNP 70, Santa Cruz sc-390899; Pol-II, abcam ab5408; PABPC, abcam ab21060; PABPN, abcam ab75855; GAPDH proteintech 60004-1-Ig; Actin, MP Biomedicals 0869100-CF; Tubulin, Sigma T9026. Western blots were visualized using an Odyssey Fc imaging system (LI-COR).

### Fractionation

Subcellular fractionation protocol was adapted from Gagnon et al., 2014, with slight modifications to buffer recipes (60). Section B, ‘Preparation of cytoplasmic, total nuclear, nucleoplasmic and chromatin fractions for biochemical assays’ was followed for total nuclear and cytoplasmic fractions. Changes to buffer recipes consisted of eliminating MgCl_2_ and adding EDTA, which helps prevent deadenylation from occurring post-lysis. Hypotonic lysis buffer consisted of 10 mM Tris, pH 7.5, 10 mM NaCl, 1 mM EDTA, 0.3% NP-40 (vol/vol) and 10% glycerol (vol/vol). Nuclear Lysis buffer consisted of 20 mM Tris, pH 7.5, 150 mM KCl, 1 mM EDTA, 0.3% NP-40 (vol/vol) and 10% glycerol (vol/vol).

### Nanopore direct RNA sequencing

RNA was prepared for nanopore direct RNA sequencing following the Oxford Nanopore Technologies (ONT) SQK-RNA002 kit protocol, including the optional reverse transcription step recommended by ONT. RNA was sequenced in-house on the minION platform using ONT R9.4.1 flow cells. Total reads (in millions) passing default filters were Cytoplasm: 2.5, Nuclear: 1.8, PABPC IP in Cytoplasm: 1.9, PABPN IP in Nucleus: 0.9.

### Nanopore direct RNA analysis

Direct RNA reads were basecalled in real time with the minKNOW software using guppy. Reads were mapped to the genome with minimap2 (v2.15) using the flags -ax splice -uf -k14 - -secondary=no. A minimap2 MAPQ score of 0 indicates multi-mapping and thus all alignments with a MAPQ score of 0 were filtered out from bam files, as well as any supplementary alignments, using samtools view -bq 1 -F 2048. Minimap2 does not currently output the typical ‘NH’ flag in bam files which indicates number of mappings per read, therefore when using featureCounts to annotate these reads, if you do not want to count multi-mappers, you must filter your bam file first because featureCounts normally would use this NH flag to determine multi-mapping. Filtered bams were used with featureCounts version 2.0.2 to annotate reads, using the flags - -minOverlap 20 and -L for long read mode. After annotation, any reads that were not from nuclear-encoded genes and mapped to the mitochondrial genome were removed from subsequent analysis and graphing.

### Poly(A) length estimation

We used the nanopolish-polya pipeline to estimate poly(A) tail lengths from basecalled reads. Reads were then filtered based on their QC tag, removing any reads that had QC tags of ‘READ_FAILED_LOAD’, ‘SUFFCLIP’ or ‘NOREGION.’

### Ribo-STAMP editing

For stable cell STAMP-fusion protein expression, cells were induced with 50 ng/ml doxycycline in DMEM for 24 h. RPS2-STAMP cell lines were used for the experimental condition and Control-STAMP (APOBEC1 only) cell lines were used for control.

### Edit distribution, EPKM and e score method details

EPKM values were determined as in (54) and metagene plots were created using metaPlotR and seaborn.

## RESULTS

### PABPN and PABPC have distinct RNA binding profiles in human cells

**Figure 1.**
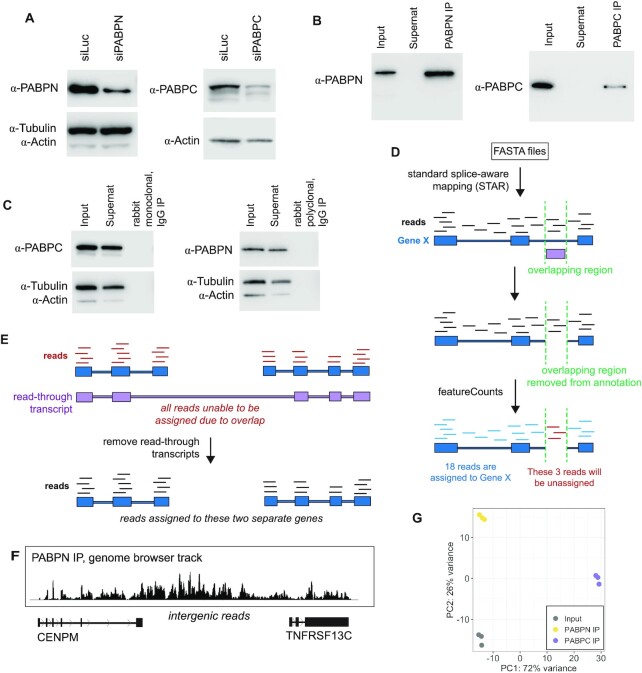
Validation of PABPN and PABPC RIPs and annotation pipeline. (**A**) Western blots showing that knockdown of PABPN or PABPC with siRNAs shows a subsequent reduction in the protein band that is targeted by the antibodies used for immunoprecipitations. Tubulin and actin are used as loading controls. (**B**) Western blots showing representative IPs pulling down PABPN or PABPC from total cell lysate. (**C**) Western blots showing matched IgG isotype control antibodies that were also used for pulldown. The RNA isolated from these RIPs was so minimal that it could not be prepared for sequencing, suggesting that the RIP conditions prevented non-specific binding of RNA. (**D**) Schematic of part of the pipeline developed to analyze RNA sequencing experiments. Any portion of the annotation file that had two or more genes overlapping the exact same genomic space was removed to avoid false positives. (**E**) Schematic showing ‘readthrough_transcript’ annotations and how they block any assignment of reads to the two genes that comprise that readthrough event. Readthrough_transcripts were removed from the annotation file. (**F**) Genome browser track read pileup for PABPN RIP showing reads that suggest failure to properly terminate transcription after the CENPM protein-coding gene, resulting in intergenic reads until reaching the neighboring gene, TNFRSF13C. (**G**) Principal Component Analysis (PCA) plot from Illumina RNA sequencing of total input, PABPC IP and PABPN IP, three independent biological replicates.

To identify the RNAs associated with PABPN and PABPC (PABPC1) at steady state, we used human total cell extract and divided this single input sample to perform RNA Immunoprecipitation (RIP) assays with either anti-PABPN or anti-PABPC. To preserve native RNA-protein interactions, HEK293T cells were crosslinked with 0.1% formaldehyde and endogenous PABPN and PABPC were immunoprecipitated along with their associated RNAs in three independent biological replicates (Figure 1A–C). Formaldehyde crosslinking was chosen so that crosslinking could be reversed with heat and the intact RNA extracted and used for total RNA sequencing (RNA-seq).

As it is known that some splicing is completed after polyadenylation, intronic reads were expected in the sequencing results (11,13). Because typical RNA sequencing (RNA-seq) analysis pipelines only quantify exonic reads, we created a pipeline that allowed quantification of reads coming from both intronic and exonic fragments. Particularly in intronic regions, current human annotation files have many overlapping genes. This presented a problem for properly annotating reads in these regions (61,62). If an RNA-seq read maps to a genomic location where multiple genes are annotated and overlapping, that read will be thrown out because it is unable to be singularly assigned. If typical pipeline parameters are changed to select one gene over another, this can unfairly bias towards exonic over intronic sections, or long genes over short genes. Additionally, there are genes present in the NCBI annotation that are not present in the gencode annotation (63,64). After noticing that our sequencing contained reads mapping to the genomic areas of these missing genes, we extracted the 672 missing genes from the NCBI annotation and manually added them to the gencode annotation. To address the issue of overlapping genes, we removed from the annotation file any regions that overlapped with two or more genes (Figure 1D). Although this functionally reduces the amount of annotated genomic space, it prevents the possibility of false positives due to mis-annotation at these regions. Furthermore, transcripts that are tagged as ‘readthrough_transcripts’ by gencode were removed as these do not represent the predominantly expressed transcript. Their presence in the annotation file causes large regions to appear overlapping when, in fact, this is a rarer event that should not preclude the ability to annotate the two individual genes comprising this annotated readthrough event (Figure 1E).

After visually inspecting the resulting reads (by converting to BigWig format and viewing on the UCSC genome browser) (65), we found that some transcripts showed evidence of improper termination and continuous downstream transcription, reminiscent of downstream-of-gene (DoG)-containing transcripts characterized previously (66–68). For example, the protein-coding gene TNFRSF13C was marked as enriched in our PABPN IP dataset, but showed reads spanning the upstream intergenic region reaching the neighboring gene CENPM (Figure 1F). The reads covering TNFRSF13C likely originated from transcripts that failed to be properly terminated from the CENPM gene. These downstream transcripts likely do not represent functional coding gene products. We filtered these genes from all analysis by quantifying the depth and breadth of reads present 2000 base pairs (bp) upstream of each annotated gene. Any genes that had coverage across 70% or more of this upstream region and an upstream expression level equivalent to 20% or more of the downstream gene were removed from results tables. This consisted of 413 genes in the input condition, 507 genes in the PABPN IP, and 124 genes in the PABPC IP (Supplementary Table S1), indicating that this type of transcription without proper termination may be more prevalent and not limited to stress conditions.

**Figure 2.**
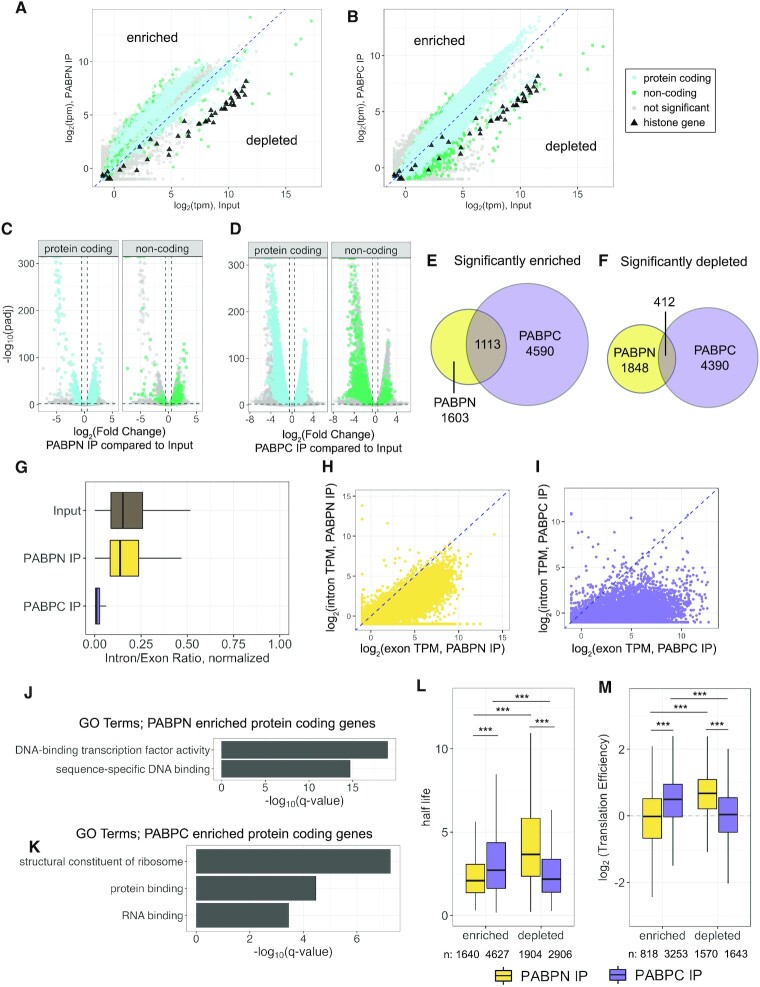
RNA Immunoprecipitation of PABPN and PABPC reveals distinct enrichment profiles. (**A** and **B**) Transcripts per kilobase million (TPM) of input compared to TPM of PABPN IP (**A**) or PABPC IP (**B**) from Illumina RNA-Seq experiments. ▴ indicates a canonical histone gene as annotated in HistoneDB 2.0 (94). Blue dashed line is an overlaid 1:1 line. Significant genes enriched or depleted were determined by comparison to input samples using DESeq2 (57) using cut-offs of log_2_FoldChange 0.5/−0.5, *P*_adj_ ≤ 0.01, and baseMean > 50. Non-significant genes are colored in grey. (C and D) Volcano plots showing enrichment and depletion of protein-coding and non-coding genes in PABPN IP vs Input (**C**) or PABPC IP versus input (**D**). Grey dashed lines indicate significance cut-offs of *P*_adj_ ≤ 0.01 and log_2_FoldChange > 0.5 or < −0.5. (**E**) Venn diagrams showing genes considered significantly enriched in PABPN IP or PABPC IP compared to input with the overlap showing the genes that were enriched in both. Cut-offs used were baseMean > 50, *P*_adj_ ≤ 0.01 and log_2_FoldChange > 0.5. (**F**) Venn diagrams showing genes considered significantly depleted in PABPN IP or PABPC IP compared to input with the overlap showing the genes that were enriched in both. Cut-offs used were the same as in (E). (**G**) Intron presence in PABPN and PABPC IPs, analyzed by normalizing each exon or intron to their respective length and then comparing the ratio of intron reads/exon reads for each gene. A value of 1 would indicate completely unspliced, and a value of 0 indicates fully spliced. Genes used for calculation had at least a TPM of 1 in both IP conditions. Box and Whisker plots show the median as the central line in the box. The upper and lower edges of the box indicate the range of the upper and lower quartiles. (**H**) Transcripts per Kilobase Million (TPM) of intronic versus exonic reads detected in PABPN IP. A pseudocount of 0.5 was added before taking the log of TPM values. Blue dashed line is an overlaid 1:1 line. (**I**) Transcripts per kilobase million (TPM) of intronic versus exonic reads detected in PABPC IP. A pseudocount of 0.5 was added before taking the log of TPM values. Blue dashed line is an overlaid 1:1 line. (**J**) Gene ontology (GO) molecular function enrichment analysis of protein coding genes significantly enriched in PABPN IP compared to Input (*n* = 1893) using PANTHER. Significance cut-offs used are baseMean > 50, *P*_adj_ ≤ 0.01 and log_2_FC > 0.5. Reference list used was the protein coding genes that were detected with a baseMean greater than 50 overall (*n* = 12 608). (**K**) Gene ontology (GO) molecular function enrichment analysis of protein coding genes significantly enriched in PABPC IP compared to Input (*n* = 5113) using PANTHER. Significance cut-offs used are baseMean > 50, *P*_adj_ ≤ 0.01 and log_2_FC > 0.5. Reference list used was the protein coding genes that were detected with a baseMean greater than 50 overall (*n* = 12 398). (**L**) Protein coding genes determined to be enriched or depleted in PABPN IP or PABPC IP were grouped and compared to published half-life values (73). Significant differences in the cumulative distributions attributable to enrichment or depletion with PABPN or PABPC are indicated: ***P < 0.001; two tailed Kolmogorov−Smirnov test. Number of genes in each boxplot are displayed at the base of the graph. (**M**) Groupings were compared to published translation efficiency data as determined by ribosome profiling (49). Otherwise, this panel is the same as in (L).

After filtering, over 15 000 genes were detected with a TPM (transcript per kilobase million) value of at least 1 in all conditions (Input, PABPN IP and PABPC IP). This confirms that the vast majority of RNAs are bound by PABPN and PABPC at some point in their lifetime. Generating a principal component analysis (PCA) plot showed that our three replicates were highly reproducible and IP conditions resulted in substantial differences, clustering separately from both input and the other IP condition (Figure 1G). To identify transcripts that were enriched or depleted from PABPN or PABPC at steady state, we used DESeq2 to compare the abundance of transcripts in IP conditions compared to total cell input (57). In this type of comparison, ‘depleted’ indicates that a transcript was detected at a lower abundance in the IP than in the input, and is not necessarily absent from the PABP IP sample. Many protein-coding genes (PCGs) and non-coding genes were enriched and depleted at steady state in both IPs (Figure 2A–D), indicating that there may be unique characteristics of an RNA that cause it to be preferentially associated with a particular PABP. Using cut-offs of log_2_FoldChange > 0.5, *P*_adj_ ≤ 0.01, and baseMean > 50, there were 2716 genes detected as significantly enriched with PABPN, and 5703 genes significantly enriched with PABPC compared to input (Figure 2E, Supplementary Table S2). 1113 of these genes were enriched in both conditions (Figure 2E, Supplementary Table S3). Using a cut-off of log_2_FoldChange < −0.5, padj ≤ 0.01, and baseMean > 50 for depletion, 2260 genes were detected as significantly depleted from PABPN and 4802 genes were significantly depleted from PABPC (Supplementary Table S2), with 412 of those genes being depleted from both conditions (Figure 2F; Supplementary Table S3).

### Transcripts of genes that encode non-polyadenylated RNAs are depleted from PABPN and PABPC RIPs

Under our stringent RIP conditions, transcripts of genes known to encode non-polyadenylated RNAs are depleted from the PABPN and PABPC RIP compared to input sequencing datasets. Replication-dependent histone genes, which terminate in a unique stem-loop structure, were depleted from both IPs but robustly detected in the input sample (Figure 2A and B). Of the 60 PCGs that are significantly depleted from both RIPs by at least 2-fold, 48 are histone genes, 2 are mitochondrial-encoded genes, and the remaining 10 are either lowly expressed or not as robustly depleted as the histone genes (Supplementary Table S3). Of the 24 lncRNAs that are two-fold depleted, 19 are from classes that would not be expected to contain a poly(A) tail, such as genes transcribed by RNA Polymerase III (Pol III), which, unlike RNA Polymerase II, is not known to associate with the nuclear polyadenylation complex (69,70). Many Pol III-transcribed genes were depleted from both RIPs but detected in the input condition, such as RNase P RNA and Y RNA (Supplementary Table S3). The remaining five lncRNAs that were robustly depleted from both RIPs are novel transcripts and their depletion suggests that they may harbor non-polyadenylated 3′ ends. Overall, the depletion of non-polyadenylated RNAs instilled confidence in our stringent immunoprecipitation conditions of these two poly(A) binding proteins.

### PABPN binds to RNAs before splicing is complete

For some introns, PABPN binding to the poly(A) tail promotes splicing. Hence, some transcripts undergo polyadenylation before intron removal has been completed (11–13). This suggests that PABPN RIP sequencing datasets could contain pre-mRNAs. To investigate whether either PABP binds to pre-mRNAs, we examined the presence of intronic sequences in the two IPs. For each gene, intronic and exonic reads were normalized to the length of the intronic or exonic region, respectively. Calculating the ratio between the two gives a value of zero if a gene is completely spliced and a ratio of 1 if normalized exonic and intronic reads are equal, thus indicating a completely unspliced gene. As predicted, transcripts associated with PABPN had a higher intron/exon ratio than when they were associated with PABPC (median PABPN ratio: 0.17, median PABPC ratio: 0.01) indicating that PABPN binds to pre-mRNA before splicing is complete (Figure 2G).

Previous reports suggest that polyadenylation preceding completion of splicing may only occur for a small subset of genes. For example, upon siRNA depletion of PABPN followed by RNA-seq, only 226 genes were observed to be >2-fold misregulated and later studies confirmed that some of these genes had difficulty splicing in the absence of PABPN (11,71). To examine whether the intron ratios shown in Figure 2G were coming from a small subset of genes, we asked how many genes had intron representation of at least 1 TPM, only considering genes that had a genomically encoded intron. Surprisingly, in PABPN RIPs, 80% of these genes contain intronic reads. In contrast, PABPC RIPs only contain intronic reads for 12% of these genes. For the 20% of genes that did not have intronic reads in the PABPN RIP dataset, their median total TPM was only 3, suggesting that the absence of intron representation for these genes may be due to their low abundance. Overall, the level of intronic reads detected in the PABPN RIP for a particular gene generally increased with the level of exonic reads (Figure 2H). In contrast, genes that were detected in the PABPC RIPs with intronic reads did not show this relationship (Figure 2I). While these results show that intron-containing transcripts for the majority of genes associate with PABPN, it is unclear if binding is dependent on the presence of a poly(A) tail or is a co-transcriptional event where PABPN is in the vicinity of the transcribing polymerase complex.

### Transcripts of genes enriched in PABPC RIPs tend to be long-lived and well-translated

Given the extent of non-overlapping RNA binding profiles (Figure 2E and F), we next asked whether there were distinguishing characteristics of the PCGs that were enriched and depleted with each PABP. To assess the molecular functions of the PCGs enriched in each IP, we used gene ontology (GO) enrichment analysis, looking at statistical overrepresentation through PantherDB (72). PCGs that encode transcripts enriched with PABPN are involved in DNA-binding, whereas those enriched with PABPC show strong enrichment for ribosomal proteins, protein binding, and RNA binding proteins (Figure 2J and K). Using published half-life values (73), we find that genes enriched with PABPC tend to encode RNAs that are more stable than those enriched with PABPN, whereas genes depleted from PABPN encode RNAs with longer half-lives than those depleted from PABPC (Figure 2L, Supplementary Table S4). This correlation is more likely due to features of long-lived RNAs that accumulate with PABPC, rather than a causative relationship between RNA stability and differential association with either PABP. Using published translation efficiency (TE) data from ribosome profiling (49), we find that PCGs enriched with PABPC and depleted from PABPN have a higher TE value, indicating that they are well-translated (Figure 2M; Supplementary Table S4). These correlations align well with what is known about the role of PABPC in facilitating translation and binding to multiple translation factors (4,74,75). Additionally, a previous study analyzing PABPC-enriched transcripts found similar positive correlations between degree of PABPC occupancy and stability and translation (76). By comparing to coding sequence (CDS) length, we find a slight positive correlation between length and degree of enrichment with PABPN PCGs (spearman 0.18, *P* < 2e−16) and a substantial negative correlation between length and degree of enrichment with PABPC PCGs (Spearman −0.59, *P* < 2e−16) (Supplementary Table S4). Genes that have been identified as encoding well-translated and highly expressed transcripts, such as housekeeping genes, are known to be more compact and have a shorter CDS length than other genes, consistent with our correlations for highly enriched PABPC PCGs (77). Altogether, these analyses indicate that transcripts of genes enriched with PABPN and PABPC differ in their average stability, translation efficiency, and coding potential.

### Ribosome contacts are higher on PCG transcripts associated with PABPC

To characterize the translation status of transcripts specifically while they are associated with PABPN or PABPC, we turned to recently developed technology that utilizes the RNA-editing enzyme APOBEC1 fused to the small ribosomal subunit RPS2, called Ribo-STAMP (Surveying Targets by APOBEC-Mediated Profiling) (54). In this system, when RPS2 is associated with an RNA (either during scanning or when complexed with the large ribosomal subunit for translation), APOBEC1 can edit that RNA in regions that are proximal to RPS2, resulting in a C to U edit that can be readily detected in Illumina RNA-seq data. A higher level of editing suggests a more well-translated substrate. Using stable HEK293T Ribo-STAMP cell lines generated by lentiviral integration (54), we performed PABPN and PABPC RIPs followed by RNA-seq. Because we are investigating the cytoplasmic process of translation, cells were fractionated and only the cytoplasmic portion was used for PABPC and PABPN RIPs. The RPS2-STAMP or control-STAMP (APOBEC1 only) constructs were induced for 24 h, the shortest time period published, at the lowest doxycycline concentration previously tested (54), in order to avoid unintended cellular effects of editing. Principal component analysis (PCA) indicated high reproducibility for the three biological replicates and no obvious effect of the STAMP constructs on separate clustering of each IP and the input samples (Supplementary Figure S1A).

**Figure 3.**
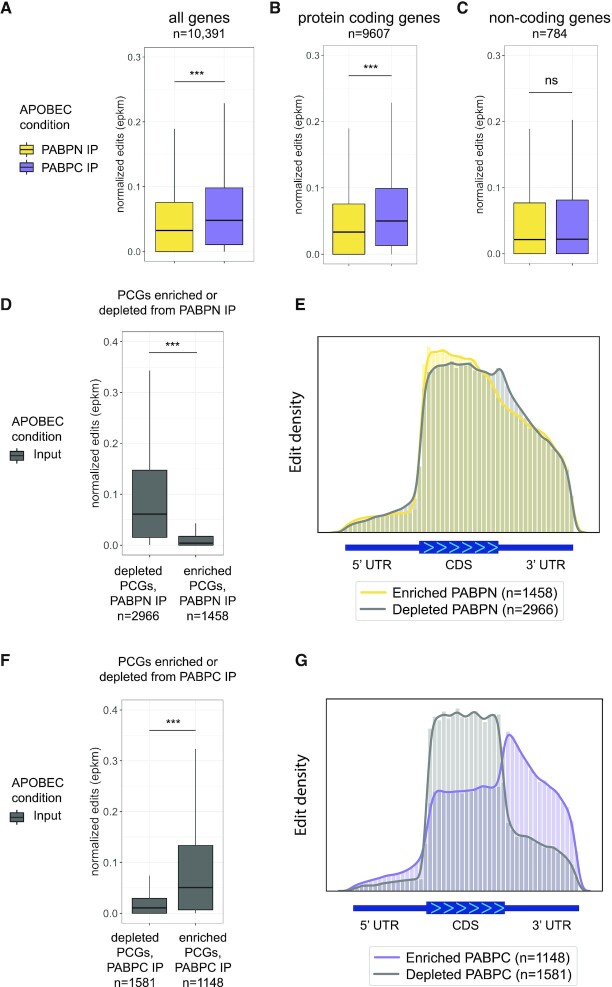
Transcripts bound to PABPC associate with the ribosome. (**A**) Boxplot of edit scores (≥0.5 confidence score) for all transcripts in PABPC IP or PABPN IP. Genes displayed have a TPM ≥ 5 in both IP conditions. ***P < 0.001; two tailed Kolmogorov–Smirnov test. (**B**) Boxplot of edit scores (≥0.5 confidence score) for protein-coding genes (TPM ≥ 5) when they are associated with PABPN or when associated with PABPC. ***P < 0.001; two tailed Kolmogorov–Smirnov test. (**C**) Boxplot of edit scores (≥0.5 confidence score) for non-coding genes (TPM ≥ 5) when they are associated with PABPN or when associated with PABPC. Significance calculated with two tailed Kolmogorov–Smirnov test. (**D**) Boxplot of edit scores (≥0.5 confidence score) from cytoplasmic input STAMP-RPS2 condition (no IP pulldown) for transcripts of protein-coding genes depleted or enriched with PABPN (TPM ≥ 5). ***P < 0.001; two tailed Kolmogorov–Smirnov test. Significance was determined by DESeq2 with cut-offs of log2FoldChange > 0.5 (enriched) or < –0.5 (depleted), baseMean > 50 and *P*_adj_ ≤ 0.01. (**E**) Metagene plot showing edit (≥0.5 confidence score) distribution for transcripts from protein-coding genes enriched or depleted from PABPN across 5′ UTR, CDS and 3′ UTR gene regions, when they were associated with PABPN. (**F**) Boxplot of edit scores (≥0.5 confidence score) from cytoplasmic input STAMP-RPS2 condition (no IP pulldown) for transcripts of protein-coding genes depleted or enriched with PABPC (TPM ≥ 5). ***P < 0.001; two tailed Kolmogorov–Smirnov test. Significance was determined by DESeq2 with cut-offs of log2FoldChange > 0.5 (enriched) or < –0.5 (depleted), baseMean > 50 and *P*_adj_ < = 0.01. (**G**) Metagene plot showing edit (≥0.5 confidence score) distribution for transcripts from protein-coding genes enriched or depleted from PABPC across 5′ UTR, CDS and 3′ UTR gene regions, when they were associated with PABPC.

Coupling Ribo-STAMP with PABPN and PABPC RIPs enabled us to study the translation status of transcripts for a given gene while associated with these different poly(A) binding proteins. We found that when transcripts are associated with PABPC, they tend to exhibit much higher levels of editing compared to when they are associated with PABPN (Figure 3A, Supplementary Table S5). While a higher degree of editing was present on PABPC-bound transcripts, many edits were also detected above background on transcripts immunoprecipitated with PABPN (Figure 3A, Supplementary Table S5). The editing that we detect when PCG transcripts are with PABPN suggests that translation is occurring while RNAs are still associated with PABPN in the cytoplasm. The PABPN RIP can potentially isolate transcripts that are coated entirely with PABPN on the poly(A) tail as well as transcripts that have both PABPN and PABPC on their poly(A) tail, as these two states are not known to be mutually exclusive (47). In either case, the higher level of editing on transcripts immunoprecipitated with PABPC suggests that the association of PABPN with translating RNAs is usually transient. The significant difference in editing due to a transcript being with PABPC remained evident when only protein-coding genes were considered (Figure 3B). However, non-coding transcripts received similarly low levels of editing when bound to either PABPC or PABPN (Figure 3C). Thus, association with PABPC alone is not sufficient for supporting high ribosome occupancy.

We next analyzed the levels and patterns of Ribo-STAMP edits on transcripts of genes enriched and depleted in the PABP IPs. Because this Ribo-STAMP experiment was focused on PABPN and PABPC in the cytoplasm, enrichment and depletion in each IP compared to the total cytoplasmic input was calculated using DESeq2. Comparisons were first made between the control-STAMP and RPS2-STAMP cell lines to determine whether inducing editing with RPS2-STAMP altered the binding profiles of PABPN and PABPC. Zero genes were found to be differentially enriched or depleted due to RPS2-STAMP induction, and therefore all samples were used together to determine enrichment and depletion for these cytoplasmic samples (Supplementary Figure S1A). Using editing status (EPKM) in the input condition, transcripts of genes depleted from PABPN have higher editing levels than those that are enriched with PABPN (Figure 3D). By plotting the position of the edits across a composite PCG, we observed a slight bias for edits towards the beginning of the coding sequence (CDS) in genes enriched with PABPN (Figure 3E). Transcripts of genes found to be enriched with PABPC had higher editing levels than those that were depleted from PABPC (Figure 3F). The pattern of edits along a composite CDS was strikingly distinct on the transcripts for genes enriched and depleted in the PABPC IPs. Genes enriched with PABPC exhibited a peak of edits near the stop codon and high levels of editing throughout the 3′ UTR, comparable to the density seen in the CDS (Figure 3G). In contrast, genes depleted in the PABPC IP accumulated edits primarily in the CDS with reduced levels in the 3′ UTR.

Considering the positive correlation between PABPC-enrichment and high levels of translation (Figure 2M and Supplementary Table S4), we asked whether this metagene profile was characteristic of well-translated genes. Graphing the top quartile of highest Ribo-STAMP edited transcripts from the input condition revealed very similar editing in the 3′ UTR compared to transcripts with the lowest quartile of editing (Supplementary Figure S1B). This suggests that a high level of translation alone does not appear to explain the editing profile of PABPC-enriched transcripts (Figure 3G compared to Supplementary Figure S1B).

We next asked whether this metagene profile was only characteristic of genes having a short CDS, as there was also a negative correlation between PABPC-enrichment and CDS size (Supplementary Table S4). Graphing separate bins of short CDS (<750 nucleotides (nt)) and long CDS genes (>750 nt) for PABPC-enriched and -depleted genes revealed that being enriched with PABPC, regardless of CDS length, was the primary indicator of high 3′ UTR editing (Supplementary Figure S1C). Although PABPC-enriched short CDS genes had a greater degree of 3′ UTR editing than PABPC-enriched long CDS genes, this analysis revealed a strikingly elevated degree of 3′ UTR editing for all PABPC-enriched genes, indicating that the presence of PABPC on a transcript was the greatest predictor of having a high proportion of editing occurring in the 3′ UTR.

To investigate whether this profile is dependent on the presence of a poly(A) tail, we graphed the replication-dependent histone genes which lack a poly(A) tail and are all in the shortest decile of genes by CDS length. Transcripts of histone genes had nearly no editing in the 3′ UTR, despite falling in this short CDS category (Supplementary Figure S1D). This indicates that the high levels of 3′ UTR editing seen for short CDS genes in Supplementary Figure S1C may be restricted to genes that have a poly(A) tail and can be bound by PABPC. Overall, these results indicate that the occupancy and access to a transcript by the ribosome is influenced by its PABP-bound state.

### Enrichment with PABPN or PABPC is related to cellular localization

**Figure 4.**
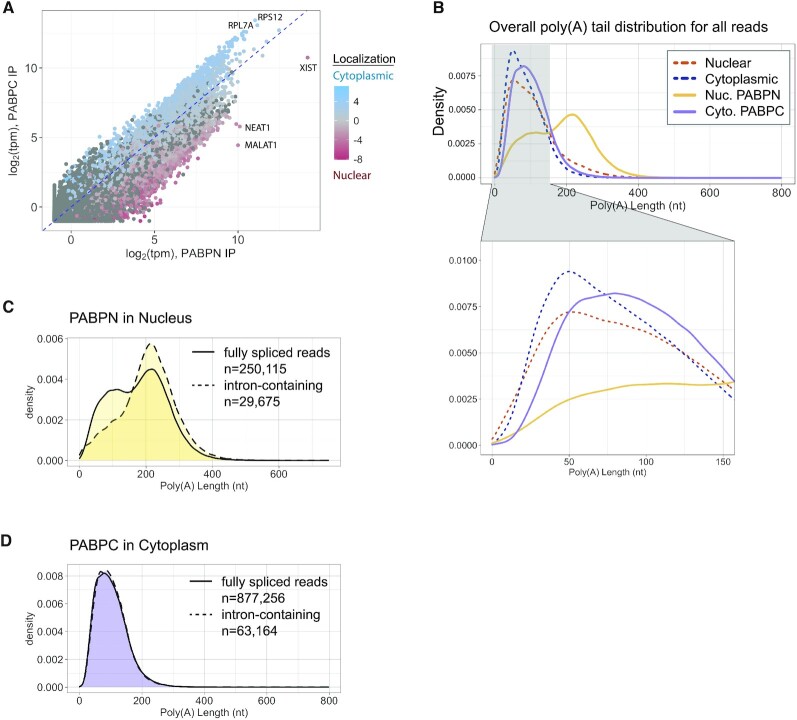
Incompletely spliced transcripts with longer poly(A) tails are associated with PABPN. (**A**) TPM of PABPN IP compared to TPM of PABPC IP, colored by degree of enrichment in cytoplasmic (blue) or nuclear (red) fractions. Select abundant genes highly enriched in the nucleus or cytoplasm are labeled by name. A pseudocount of 0.5 was added before taking the log of TPM values. Only genes with mature length >200nt are plotted, as PABPC and PABPN rarely bind to shorter transcripts. Replication-dependent histone genes (which are depleted from both IPs) were used as a threshold for background in the IP conditions and any gene that was detected at a lower ratio than these histone genes was not included in this plot. (**B**) Density plot showing overall poly(A) tail length distribution of all nuclear-encoded genes detected by Nanopore direct RNA sequencing. Dashed lines indicate total cytoplasm and total nuclear fraction, and solid lines indicate the two IP conditions. Region from 0–150 nt is magnified in the bottom panel to show the differences in distributions for shorter poly(A) tails. Number of reads in each density plot line are as follows: Nuclear 585 914; Cytoplasm 1 039 310; Nuclear PABPN 300 565; Cytoplasm PABPC 1 047 845. (**C**) Density plot of poly(A) tail length for reads that contained one or more introns (dashed line) or no introns (solid line) while with PABPN in the nucleus. (**D**) Density plot of poly(A) tail length for reads that contained one or more introns (dashed line) or no introns (solid line) while with PABPC in the cytoplasm.

Considering the predominant localization of PABPN in the nucleus and PABPC in the cytoplasm (39,78), we predicted that RNAs enriched with each protein would similarly partition. First, we plotted the TPM values for transcripts of genes when associated with PABPN versus when associated with PABPC, which revealed a strong bias of many transcripts for associating with one protein more than the other (Figure 4A). To determine whether these profiles correlate to biased localization of an RNA either in the nucleus or the cytoplasm, we performed subcellular nuclear and cytoplasmic fractionation and sequenced the RNAs in each compartment (Supplementary Figure S2A and B). By comparing relative RNA localization to the abundance of that RNA in each of the RIPs, we observed that RNA localization largely reflects enrichment with PABPN or PABPC (Figure 4A, Supplementary Table S6). RNAs that are highly cytoplasmic tend to be more associated with PABPC than with PABPN, and RNAs that are more nuclear restricted tend to be enriched with PABPN. As expected, the nuclear localized non-coding RNAs XIST, NEAT1 and MALAT1 show a much greater association with PABPN (Figure 4A). In contrast, highly stable transcripts, such as those encoding ribosomal proteins, predominantly exist in the cytoplasm bound to PABPC (Figure 4A).

### Poly(A) tails are longer when a transcript is associated with PABPN

Previous poly(A) tail analyses concluded that tails are longest in the nucleus and undergo shortening over time once they are in the cytoplasm (16,79). The degree of difference reported in these early studies for nuclear and cytoplasmic steady-state poly(A) tail sizes was relatively minimal. For instance, work in HeLa cells showed a 30 nt difference between the predominant peak of tail sizes found in the nucleus versus the cytoplasm (16). More recent work using 5-ethynyl uridine (5EU) time-course labeling in mouse 3T3 cells was able to capture a greater difference between newly made and steady state tails, potentially due to this specific time-course labeling and high-throughput sequencing (52). Although they note great inter- and intragenic variation for tail length, they found the peak of distributions for newly made tails centers around 175 nt long, while steady state centered around 100 nt (52). Considering the model of sequential binding of PABPN to nascent transcripts followed by PABPC as they undergo translation (26), we asked if the RNAs bound by these proteins had different poly(A) tail lengths.

Using subcellular fractionated extracts, we performed RIP of PABPN from the nucleus and PABPC from the cytoplasm and used Nanopore direct RNA sequencing to interrogate poly(A) tail length on transcripts when they were associated with these proteins (Supplementary Figure S2A and B, Table S7). Direct RNA sequencing has the distinct advantage of being able to sequence the entire transcript as well as using pore dwell time to infer poly(A) tail length (51,80). Consistent with previous reports (16,52,79), a greater fraction of long poly(A) tail reads were observed for total nuclear versus cytoplasmic RNAs (Figure 4B). Close examination of the shortest detected tail sizes showed that tail sizes less than 15 nt were detected in the total nuclear and cytoplasmic RNA but were largely nonexistent in the IP samples. This observation is consistent with a minimal poly(A) tail size greater than ∼12 being needed for stable association with PABPN or PABPC *in vivo*. Previous studies using Illumina-based sequencing to examine poly(A) tails of PABPC-associated transcripts also found a lack of very short poly(A) tails on transcripts that were associated with PABPC, despite those very short tails being detected in the input condition (76). Most striking, though, was the difference in tail lengths of RNAs associated with PABPN in the nucleus compared to all other samples, including the total nuclear fraction (Figure 4B). These results suggest that association with PABPN may be a prerequisite for the maintenance of poly(A) tail sizes over 200 nt.

Since we detected intronic reads associated with PABPN and, to a much lesser extent, PABPC (Figure 2G), we took advantage of the full length reads generated by Nanopore direct RNA sequencing to further examine the pre-mRNAs bound by these proteins. When reads were separated based on whether they contained introns or not, we observed distinct poly(A) tail size profiles for the PABPN-bound transcripts. Transcripts that still had at least one intron present tended to have a relatively uniform longer poly(A) tail distribution that centered around 230 nt (Figure 4C). In contrast, fully spliced transcripts produced a peak around 230 nt as well as a broad shoulder of shorter tail sizes (Figure 4C). To investigate whether this was due to an inherent difference in the types of genes in these categories, we only analyzed genes that had a representative intron-containing read and observed comparable profiles (Supplementary Figure S2C). These patterns seen in poly(A) tail length distribution based on intron presence likely account for the phased profile of all tail sizes on transcripts bound to PABPN seen in Figure 4B. The retention of an intron in the PABPC-bound transcripts did not seem to influence poly(A) tail length, as fully spliced and intron-containing transcripts exhibited nearly identical distributions that centered around 80 nt (Figure 4D). Taken together, these analyses show that in the nucleus, PABPN can associate with pre-mRNAs and that poly(A) tail sizes can reach maximum lengths prior to completion of splicing.

**Figure 5.**
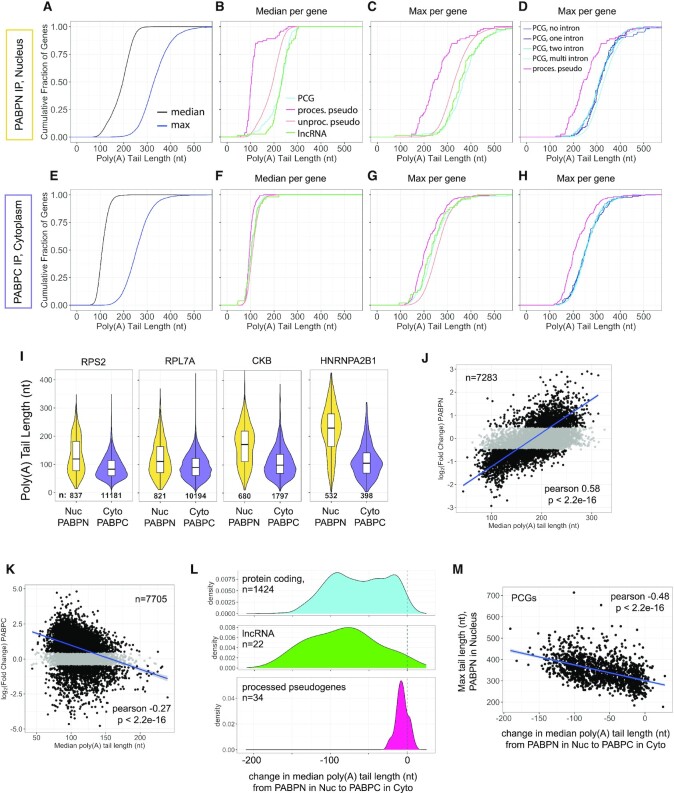
Poly(A) tail size differs depending on whether a transcript is associated with PABPN or PABPC. (**A**) Cumulative plot showing median and maximum poly(A) tail length for each gene that had at least 10 reads in PABPN IP from the nucleus (*n* genes = 7343). Graphing area for all cumulative plots has been limited to 0–550 nt. (**B**) Cumulative plot showing median tail length detected in PABPN IP in the nucleus, separated by gene biotype, for genes that had at least 10 reads. PCG *n* = 7025, processed pseudogene *n* = 73, unprocessed pseudogene *n* = 72, lncRNA *n* = 168. (**C**) Cumulative plot showing maximum tail length detected in PABPN IP in the nucleus, separated by gene biotype, for genes that had at least 10 reads. PCG *n* = 7025, processed pseudogene *n* = 73, unprocessed pseudogene *n* = 72, lncRNA *n* = 168. (**D**) Cumulative plot showing maximum tail length detected in PABPN IP in the nucleus for protein coding genes that contain none, one, two or multiple introns, as well as processed pseudogenes, for genes that had at least 10 reads. PCG no introns *n* = 104, PCG 1 intron *n* = 140, PCG 2 introns *n* = 217, PCG 3 + introns *n* = 6563, processed pseudogene *n* = 72. (**E**) Cumulative plot showing median and maximum poly(A) tail length for each gene that had at least 10 reads in PABPC IP from the cytoplasm (*n* genes = 7736). (**F**) Cumulative plot showing median tail length detected in PABPC IP from the cytoplasm, separated by gene biotype, for genes that had at least 10 reads. PCG *n* = 7209, processed pseudogene *n* = 236, unprocessed pseudogene *n* = 52, lncRNA *n* = 233. (**G**) Cumulative plot showing maximum tail length detected in PABPC IP from the cytoplasm, separated by gene biotype, for genes that had at least 10 reads. PCG *n* = 7209, processed pseudogene *n* = 236, unprocessed pseudogene *n* = 52, lncRNA *n* = 233. (**H**) Cumulative plot showing maximum tail length detected in PABPC IP from the cytoplasm for protein coding genes that contain none, one, two or multiple introns, as well as processed pseudogenes, for genes that had at least 10 reads. PCG no introns *n* = 134, PCG 1 intron *n* = 209, PCG 2 introns *n* = 308, PCG 3 + introns *n* = 6557, processed pseudogene *n* = 236. (**I**) Violin plots of individual genes and their poly(A) tail distributions when associated with PABPN in the nucleus and when associated with PABPC in the cytoplasm. RPS2 and RPL7A are among the top most abundant genes in both PABPN IP from the nucleus and PABPC IP from the cytoplasm. CKB and HNRNPA2B1 are among the top most abundant with PABPN in the nucleus. Number of reads for each violin are shown in black text at the base of the violin. White boxplots are inlaid within the violin, indicating the median (line) and upper and lower quartiles (box). The lines extending out from the central box indicate the minimum and maximum value in that dataset. (**J**) Median poly(A) tail length when transcripts were associated with PABPN compared to their degree of enrichment or depletion as determined by total cell lysates and comparison of PABPN IP to input condition. Genes had to have at least 10 poly(A) reads to be considered in this analysis. Blue line is a best fit line using a linear model. Data points in black are genes that are significantly enriched/depleted, determined as previously in Figure 1, using cutoffs of *P*_adj_ ≤ 0.01, baseMean > 50 and log_2_FC of > 0.5 or < −0.5. (**K**) Median poly(A) tail length when transcripts were associated with PABPC compared to their degree of enrichment or depletion as determined by total cell lysates and comparison of PABPC IP to input condition. Cut-offs are the same as described in (J). (**L**) Density plots of the change in median poly(A) tail length when a transcript is with PABPN in the nucleus compared to PABPC in the cytoplasm, separated by gene biotype. A negative change indicates that the poly(A) tail was shorter when associated with PABPC in the cytoplasm. Genes must have been represented by at least 35 reads in each IP to be displayed. Unprocessed pseudogenes did not have enough reads to pass cut-offs and are thus not displayed here. (**M**) Change in median poly(A) tail length of all protein coding genes that were displayed in blue in (L), compared to their maximum detected poly(A) tail length when associated with PABPN in the nucleus. Blue line is a best fit line using a linear model.

To determine whether the dramatic difference in poly(A) tail sizes detected on transcripts associated with PABPN versus PABPC reflected the entire population or more limited sets of abundant transcripts (Figure 4B), we calculated the median and maximum tail lengths for each gene (Figure 5A-H, Supplementary Table S7). Transcripts for 46% of all genes bound to PABPN had median poly(A) tail sizes over 200 nucleotides long, with a range of 43–325 nt (Figure 5A). In contrast, the median for all genes with transcripts bound to PABPC was 108 nt, with a range of 42–239 nt (Figure 5E) (*P*-value < 2e–16, two tailed Kolmogorov–Smirnov test comparing median poly(A) tail length when with PABPN or PABPC). Overall, most genes exhibited much longer poly(A) tail sizes on transcripts bound to PABPN compared to PABPC. This is exemplified in Figure 5I where we plotted the tail length for all transcripts of a given gene associated with PABPN or PABPC (Figure 5I). The transcripts for the ribosome protein-encoding genes, RPS2 and RPL7A, are among the most abundant detected in RIPs for both PABPN and PABPC and exhibit distinct poly(A) tail size profiles when associated with each protein. The median and average tail lengths are shorter on transcripts associated with PABPC (Figure 5I). This difference is exacerbated for genes with high numbers of transcripts bound to PABPN, such as CKB and HNRNPAB1 (Figure 5I).

### Transcripts with poly(A) tails longer than 200 nt are bound by PABPN and PABPC

While the median poly(A) tail size largely differed when transcripts for a given gene were associated with PABPN (196 nt) versus PABPC (108 nt), much longer tailed representatives were isolated with both PABPs. The maximum poly(A) tail size was typically over 200 nucleotides for transcripts of a given gene when associated with PABPN or, unexpectedly, PABPC (Figure 5A and E). The maximum tail length for genes detected with at least 10 reads in the PABPN IP was >200 nt for >99% of genes (Figure 5A). This is consistent with earlier *in vitro* polyadenylation and radioactive studies, which demonstrated that the majority of newly-made poly(A) tails are ∼200 adenosines long (16,17). With PABPC in the cytoplasm, >86% of genes have a transcript with a tail length >200 nt, suggesting that shortening of the poly(A) tail may occur after the RNA reaches the cytoplasm and acquires PABPC on its poly(A) tail (Figure 5E). Although max poly(A) tail sizes were longer than 200nt for most transcripts bound by either PABPN or PABPC, the greatest max tail sizes were seen when transcripts were bound by PABPN (*P*-value < 2e−16, two tailed Kolmogorov–Smirnov test comparing maximum poly(A) tail length when bound by PABPN or bound by PABPC).

When the median and maximum poly(A) tail length values were separated by gene biotype, processed pseudogenes stood out as a class exhibiting much shorter tail lengths than other biotypes when associated with PABPN (Figure 5B and C). This difference was much less evident when transcripts for processed pseudogenes were associated with PABPC (Figure 5F and G). Processed pseudogenes typically lack introns as they are thought to have undergone retrotransposition from a mature RNA, rather than a duplication event which occurs with unprocessed pseudogenes (81). Therefore, we asked whether lacking introns might be related to the shorter median and maximum tail lengths observed for processed pseudogenes by separating PCGs into those with no encoded introns, one intron, two introns or multiple introns (three or more encoded in the genome). None of these categories match the distinctly shorter length of processed pseudogenes with PABPN, indicating that the presence of introns and a need for splicing is not necessary for acquiring a maximum tail length greater than 200 nt (Figure 5D and H).

Using the differential enrichment and depletion data generated from our total cell lysate and IP conditions (Figure 2), we correlated median poly(A) tail length of a given gene while associated with PABPN or PABPC to degree of enrichment with that particular PABP. While associated with PABPN, median poly(A) tail length had a positive correlation on transcripts of genes found to be enriched with PABPN (Figure 5J). On the other hand, while associated with PABPC, median poly(A) tails showed a negative correlation with enrichment with PABPC (Figure 5K).

For all gene biotypes, the median poly(A) tail lengths were substantially shorter for transcripts of a given gene bound by PABPC compared to PABPN and different genes showed varied degrees of shortening (Figure 5I, Supplementary Figure S3A and B). The extent of shortening is stunted for processed pseudogenes due to their shorter initial poly(A) tail lengths (Figure 5L). The initial poly(A) tail size, inferred as the maximum tail length for a gene bound by PABPN, varied greatly for protein coding genes (range: 125–713 nt) and the longer the initial tail length, the greater shortening that gene underwent after association with PABPC (Figure 5M). This difference suggests that substantial pruning of the poly(A) tail occurs as a transcript transitions from binding PABPN to PABPC. Altogether, these analyses show that distinct populations of RNAs, differentiated by splicing status and poly(A) tail length, are present with PABPN versus PABPC.

## DISCUSSION

The prevailing model of PABP association suggests that for the majority of RNAs, PABPN binds nascent RNAs in the initial stages of polyadenylation and facilitates creation of the poly(A) tail. Upon transport to the cytoplasm, PABPN is replaced by PABPC on the poly(A) tail, which promotes translation and stability of the RNA. Here, we examine several aspects of this model by performing comprehensive and direct RNA sequencing of RNAs bound by PABPN and PABPC in human cells. While almost all known polyadenylated transcripts were detected in both the PABPN and PABPC IPs, the relative amount differed substantially for many RNAs. Consistent with the model, transcripts enriched with PABPN tended to have biased nuclear localization and hallmarks of nascent RNAs, such as intronic reads and long poly(A) tails. In contrast, transcripts enriched with PABPC tended to be more efficiently translated and longer-lived with pruned poly(A) tails. Moreover, our analyses raised new considerations for the timing and roles of PABPN and PABPC in their association with a given RNA from its synthesis to its functional state.

### PABPN associates with mRNAs prior to completion of splicing

While PABPN is known to bind nascent transcripts, the timing of PABPN loading and factors that affect association are poorly described. In this work we present evidence that PABPN associates early in the life of an mRNA, before splicing is complete. Due to the nature of crosslinking and RIP, this association may be a direct association (bound directly to the RNA) or it may be that PABPN was very immediately nearby, perhaps tracking with the transcribing polymerase. In our RIP sequencing data, we find that intron containing species are present for 80% of genes represented. This may point to a mechanism where PABPN is loaded co-transcriptionally, a notion that is supported by some evidence indicating association of PABPN with RNA Pol II in insects (82) and evidence of PABPN’s ability to influence alternative polyadenylation selection (83). This evidence suggests that polyadenylation can begin and possibly complete before splicing is finished, a model that is also supported by our nanopore sequencing data showing that intron containing mRNAs tend to have very long poly(A) tails (Figure 4C). The distinct poly(A) tail lengths between intron-containing mRNAs and fully spliced mRNAs also suggests that the completion of splicing may limit poly(A) extension. Interestingly, we find that intron-containing transcripts associated with PABPC show no difference in tail size compared to the fully spliced transcripts. This could be due to unannotated splice variants, but because the majority of these genes only have one intron-containing read in our sequencing data, we believe that it's more likely that these are defective splicing products.

### Distinct poly(A) tail profiles are associated with PABPN and PABPC

Recent genome-wide studies have discovered that steady state poly(A) tail length is not uniform for all transcripts of a given gene and median tail sizes for each gene vary considerably (49,84–87). Most of these studies have captured a singular distribution of poly(A) size for each gene, taking a snapshot of the entire cell at once. Eisen et al. performed a time-course to investigate cytoplasmic poly(A) tail lengths, showing that greater variation can be captured this way (52). Here we show that part of this variation results from distinct poly(A) tail profiles of RNAs associated with PABPN versus PABPC. By isolating each endogenous protein from the same cell extract, we were able to make direct comparisons between PABPN- and PABPC-bound transcripts. Consistent with the time course study showing longer poly(A) tails on newly synthesized transcripts (52), we found that RNAs bound by PABPN tended to have much longer poly(A) tails than those bound by PABPC. The striking difference in poly(A) tail profiles for RNAs isolated with PABPN compared to nuclear extract suggests that PABPN IP selects for a subset of nuclear RNAs that are obscured in the total nuclear sample. From this population of RNAs captured by PABPN, we were able to determine that virtually all polyadenylated genes achieve maximal poly(A) tail lengths of at least 200 nucleotides and many can be hundreds of nucleotides longer. Illumina-based sequencing approaches measuring the poly(A) tail have been unable to accurately quantify very long tails. For TAIL-seq, the maximum possible detected poly(A) tail is 230 nt (50,86,88), and for PAL-seq, poly(A) standards up to ∼300 adenosines were used to calculate the linear regression (49). Similar to our studies here, previously published Nanopore sequencing also detected poly(A) tails up to ∼600 adenosines in human cells (51). The functional consequences of having a poly(A) tail longer than 200–300 nt have not been investigated and may have implications for downstream interactions.

When associated with PABPN in the nucleus, transcripts for a given gene showed a range of tail sizes, rather than clustering specifically at a uniform tail size (Figure 5I). Interestingly, a few genes had transcripts that exhibited tail sizes of >400 nt (Figure 5A). However, in all cases, this represented a small fraction of the total poly(A) reads. One gene that stood out was XIST (X-inactive specific transcript), which had 224 transcripts captured with tail lengths >400 nt. This comprised only 8% of the total reads for this gene but was the highest number of extremely long-tailed transcripts of a specific gene seen in our dataset. As XIST remains associated with the chromosome from which it was transcribed (89), this raises the question of whether chromatin retention influences termination of polyadenylation.

A notable exception to these described poly(A) trends are transcripts of processed pseudogenes, which displayed strikingly short tails regardless of whether they associated with PABPN or PABPC. Because processed pseudogenes are thought to originate from a mature mRNA being inserted back into the genome via retrotransposition, processed pseudogenes do not have introns (81). For many genes, splicing and polyadenylation is thought to occur at nearly the same time, so one possible explanation for their extremely short tails is that the presence of splicing machinery on a transcript may be necessary for proper full polyadenylation. However, looking at the few intron-less protein-coding genes, we found that these examples do not have the same short tail phenotype. This would suggest that at least in this context, the splicing machinery is not necessary for proper polyadenylation.

Interestingly, maximum tail lengths >200 nt were found for the majority of genes whether a transcript was associated with PABPN or PABPC. This supports a model where tails can reach a length of at least 200 adenosines when they are associated with PABPN in the nucleus and subsequent shortening of the RNA, termed pruning, largely occurs after a transcript is bound by PABPC in the cytoplasm. The functional implications of this process and why a cell would expend energy to produce a long poly(A) tail just to shorten it later are not understood. Evidence supporting tail shortening exists in many organisms, suggesting that pruning may be a coordinated, conserved process (49,86). Recent studies of deadenylases that delineated their precise functions in the presence of PABPC have provided insights into some of the players (84,85), but many mechanistic details remain to be discovered. Furthermore, our RIP experiments differentiate transcripts based on PABP partners but for a given PABP, we cannot distinguish between RNAs of different ages. Future time-course studies could elucidate whether the tail length variation observed within the PABPC dataset or the PABPN dataset is due to aging of the RNA or intragenic variation. Additionally, the examination of different cell types and cells grown under different conditions is likely to reveal distinct RNA profiles bound to PABPN and PABPC.

### Ribo-STAMP reveals differences in translation status for PABPN- and PABPC-bound transcripts

Using the recently developed Ribo-STAMP technique, we sought to infer the translation status of transcripts bound to either PABPN or PABPC. Many experiments that infer translation status give a singular readout as an average of all the transcripts in the cell. However, Ribo-STAMP enabled us to examine the translation status of an mRNA after subsetting by its association to PABP (by crosslinking RIP). PCGs bound by PABPC showed the highest levels of editing which would indicate relatively higher levels of association with translation machinery and greater translation efficiency. Unexpectedly, genes that were enriched with PABPC showed a high proportion of editing occurring in the 3′ UTR. While release of the ribosome from mRNA upon recognition of the STOP codon should limit its contact with the 3′ UTR, this phenomenon has been documented by other ribosome-occupancy methods, including the original Ribo-STAMP methods paper (54,90–93). When binned by CDS length for PABPC-enriched and -depleted genes, we observed that enrichment with PABPC was the greatest indicator of high 3′ UTR editing, not CDS length. Further emphasizing the importance of PABPC binding for this 3′ UTR metagene profile, replication-dependent histone genes, which contain short coding sequences and terminate in a stem-loop instead of a poly(A) tail, showed little to no editing in the 3′ UTR. The connection between ribosome access to the 3′ UTR, CDS length, and the presence of a poly(A) tail suggests the possibility of a conformational state that is facilitated by PABPC and leads to a greater proximity of translating ribosomes with the 3′ UTR. Editing of the 3′ UTR seen in Ribo-STAMP and similar ribosome occupancy methods may reflect a meaningful biological state of particular mRNAs, where transcript size and PABPC occupancy contribute to a conformation that may, for example, promote ribosome recycling.

Overall, our findings provide a genome-wide view of the identities, splicing status, ribosome occupancy, and polyadenylation state of RNAs that preferentially associate with PABPN or PABPC in human cells. An important and broadly applicable conclusion of this work is that most RNAs exist as a heterogenous pool, partly distinguished by being bound to PABPN or PABPC, and thus, may be differentially susceptible to specific post-transcriptional regulatory mechanisms. Read-outs of regulation such as changes in steady state mRNA levels or poly(A) tail length may gain sensitivity if specific PABP-bound transcripts are considered instead of the entire cell population.

## DATA AVAILABILITY

RNA-seq data are available through the Gene Expression Omnibus (GEO) series accession GSE195493, GSE195494, and GSE195495.

## Supplementary Material

gkac263_Supplemental_Files
