# Supplementary material for: Nuclear and cytoplasmic poly(A) binding proteins (PABPs) favor distinct transcripts and isoforms
Source: Nucleic Acids Res. 2022 Apr 19;50(8):4685–702. doi: 10.1093/nar/gkac263 (PMC9071453; doi:10.1093/nar/gkac263)
Supplement: gkac263_Supplemental_Files [file gkac263_supplemental_files.zip › Supplemental-Nicholson-Shaw.pdf]

Supplemental Figure 1

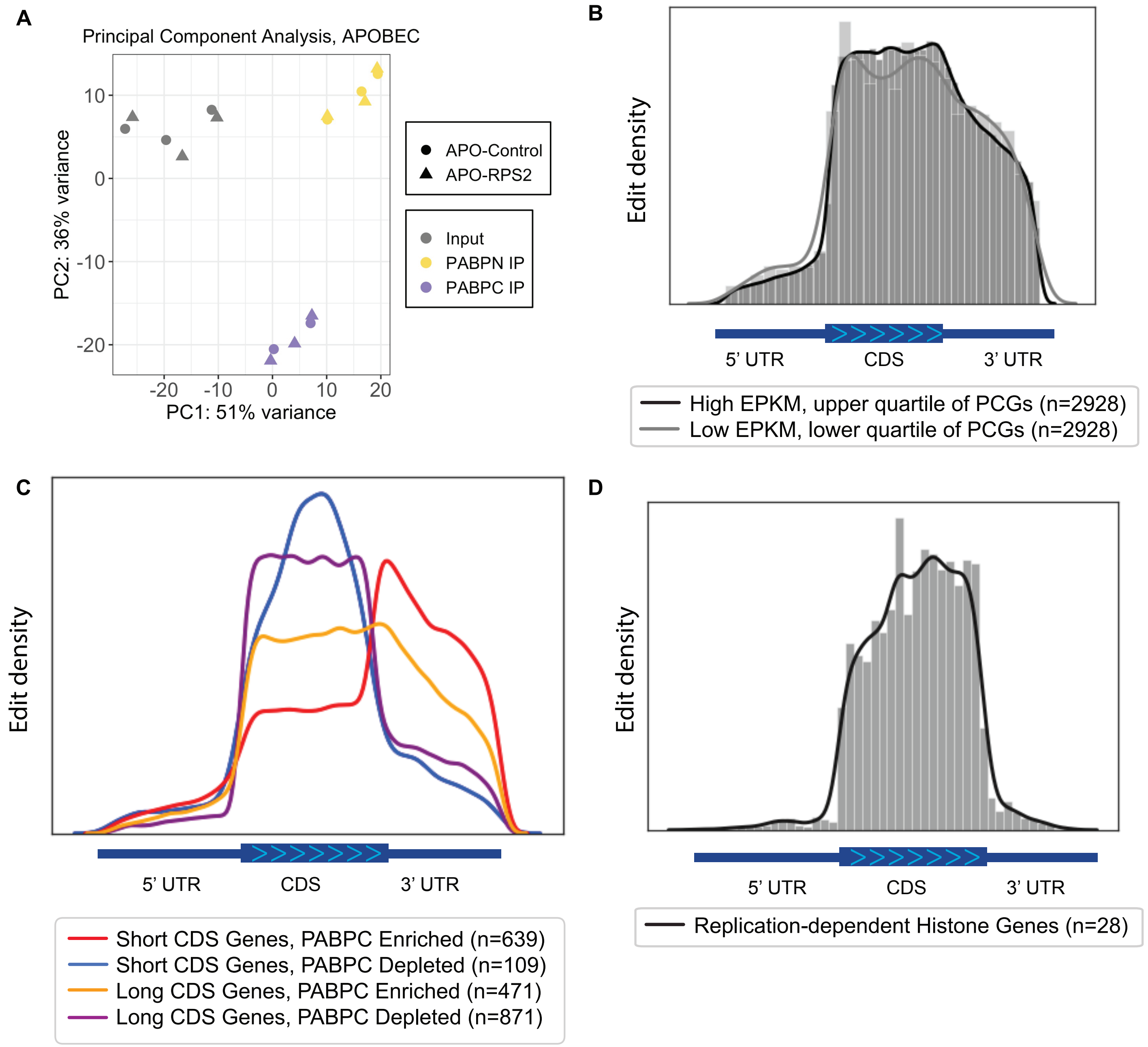

Supplemental Figure 2

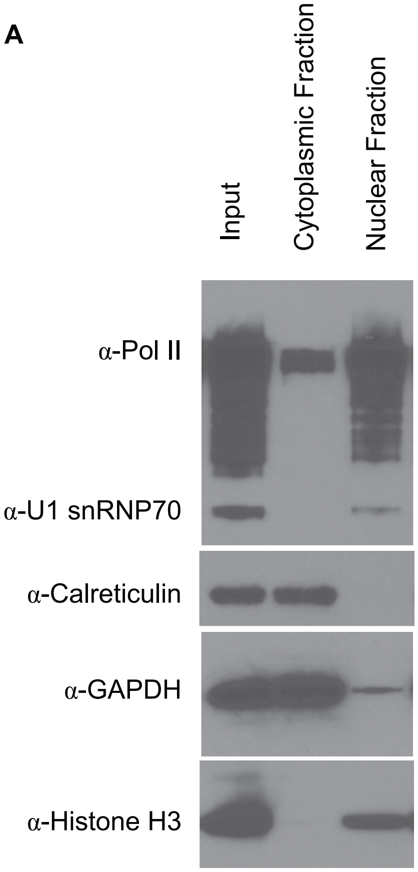

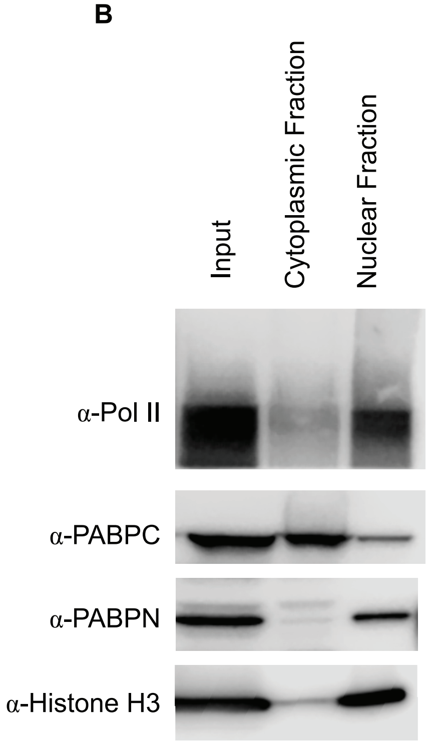

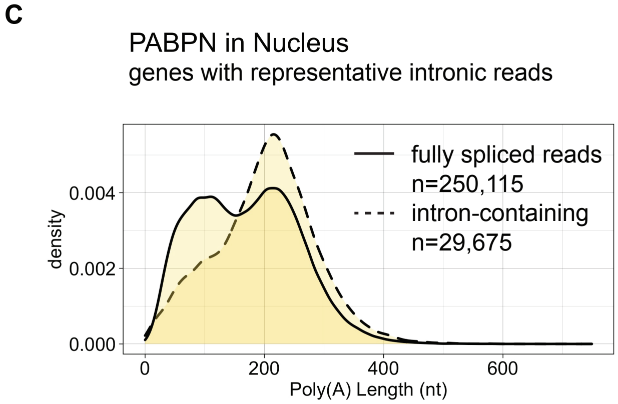

Supplemental Figure 3

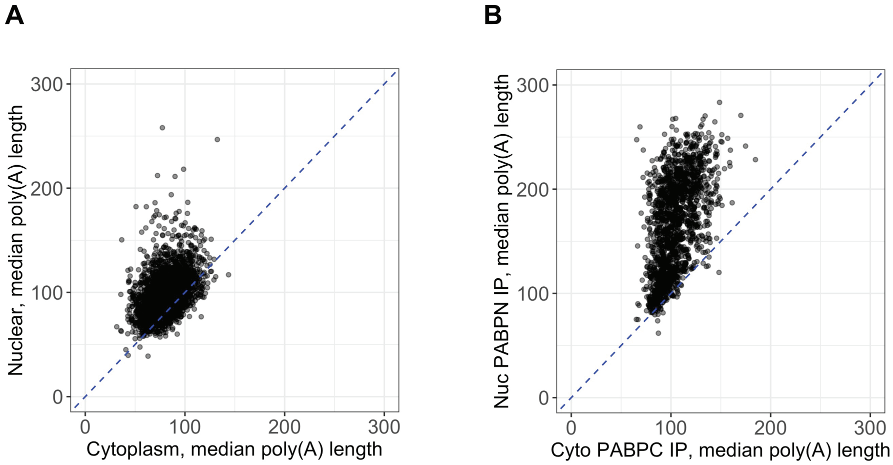

SUPPLEMENTAL FIGURE LEGENDS

Supplemental Figure 1: Ribosome contact with transcripts inferred by Ribo-STAMP. (A) Principal Component Analysis plot from Illumina RNA sequencing of Control-STAMP and RPS2-STAMP cell lines. (B) Metagene plot showing edit (>=0.5 confidence score) distribution for transcripts of protein-coding genes that were in the upper quartile for editing (highly translated) compared to the lowest quartile for editing (lowly translated) from the input condition (no IP pulldown). (C) Metagene plot showing edit (>=0.5 confidence score) distribution for transcripts of protein-coding genes separated by CDS length (short CDS genes are < 750 nt in CDS, and long CDS are > 750 nt) from the PABPC IP condition. (D) Metagene plot showing edit (>=0.5 confidence score) distribution for transcripts of replication dependent histone genes.

Supplemental Figure 2: **Nuclear and cytoplasmic distribution of PABPN and PABPC** (A) Representative western blot of subcellular fractionation. (B) Western blot showing the nuclear and cytoplasmic distribution of PABPN and PABPC (C) Density plot of poly(A) tail length for reads that contained one or more introns (dashed line) or no introns (solid line) while with PABPN in the nucleus, only for genes that had an intron-containing representative.

Supplemental Figure 3: Poly(A) tails are longer on transcripts in the nucleus and with PABPN. (A) Median poly(A) tail length (per gene) in the cytoplasm compared to median poly(A) tail length in the nucleus. Genes must have been represented by at least 35 reads in each condition to be displayed. (B) Median poly(A) tail length (per gene) in PABPN IP from the nucleus compared to median poly(A) tail length in PABPC IP from the cytoplasm. Genes must have been represented by at least 35 reads in each condition to be displayed.

SUPPLEMENTAL TABLES

Supplemental Table 1: Genes with problematic upstream transcription that were removed from further analysis

Supplemental Table 2: Significantly enriched and depleted genes for PABPN and PABPC RIPs compared to input condition.

Supplemental Table 3: Genes Enriched and Depleted in Common from both PABPN and PABPC RIP

Supplemental Table 4: Correlations for protein coding genes that are enriched or depleted from PABPN IP or PABPC IP

Supplemental Table 5: EPKM values determined by Ribo-STAMP experiments. Background editing detected in control conditions was subtracted to obtain normalized EPKM values for each gene. All edits used had >= 0.5 confidence score.

Supplemental Table 6: TPM values and log2FoldChange of cytoplasmic or nuclear distribution used to generate Figure 3A. Log2FoldChange values were determined by subcellular fractionation and RNA-seq, using cut-offs for significance of padj <=0.01. Negative indicates a

gene was more enriched in the nucleus and positive indicates a gene was more enriched in the cytoplasm.

Supplemental Table 7: Poly(A) tail lengths as determined by Nanopore direct RNA sequencing for total cytoplasmic input, total nuclear input, PABPC IP from the cytoplasm, and PABPN IP from the nucleus.
